# Temporal binding as multisensory integration: Manipulating perceptual certainty of actions and their effects

**DOI:** 10.3758/s13414-021-02314-0

**Published:** 2021-06-01

**Authors:** Annika L. Klaffehn, Florian B. Sellmann, Wladimir Kirsch, Wilfried Kunde, Roland Pfister

**Affiliations:** Department of Psychology III, University of Würzburg, Röntgenring 11, 97070 Würzburg, Germany

**Keywords:** Perception and action, Multisensory processing, Temporal processing

## Abstract

It has been proposed that statistical integration of multisensory cues may be a suitable framework to explain temporal binding, that is, the finding that causally related events such as an action and its effect are perceived to be shifted towards each other in time. A multisensory approach to temporal binding construes actions and effects as individual sensory signals, which are each perceived with a specific temporal precision. When they are integrated into one multimodal event, like an action-effect chain, the extent to which they affect this event’s perception depends on their relative reliability. We test whether this assumption holds true in a temporal binding task by manipulating certainty of actions and effects. Two experiments suggest that a relatively uncertain sensory signal in such action-effect sequences is shifted more towards its counterpart than a relatively certain one. This was especially pronounced for temporal binding of the action towards its effect but could also be shown for effect binding. Other conceptual approaches to temporal binding cannot easily explain these results, and the study therefore adds to the growing body of evidence endorsing a multisensory approach to temporal binding.

**Supplementary Information:**

The online version contains supplementary material available at 10.3758/s13414-021-02314-0.

## Introduction

In their everyday life, most humans are bombarded with perceptual information, most of it redundant and irrelevant. When faced with such abundance, one way to cope lies in multisensory integration, which links related sensory signals and thereby helps us perceive coherent multimodal events rather than a host of independent sensory signals. This process forms a cornerstone of everyday perception, and its prevalence is documented by striking multisensory illusions such as the ventriloquist effect (Alais & Burr, 2004). Here, concurrent visual and auditory signals are merged into an integrated percept by fusing their perceived location. However, integration is not limited to the spatial dimension, but also affects other attributes such as the intensity (Stein et al., 1996) and the perceived timing of stimuli (Fendrich & Corballis, 2001; Shams, Ma, & Beierholm, 2005). Despite these long-known insights, theories of multisensory integration have only recently been applied to the phenomenon of temporal binding, a perceptual illusion in the temporal domain (Cao et al., 2020; Kirsch et al., 2019; Wolpe et al., 2013).

Temporal binding – or *intentional binding* as it was termed when first described (Haggard et al., 2002) – occurs when two causally related events are perceived as shifted toward each other in time. Initially, temporal binding was proposed to arise from predictive mechanisms in intentional, voluntary motor actions (*motor approach*). Therefore, the illusion has received particular interest in research on how human agents perceive the consequences of their own actions, and intentional binding has often been used as a proxy for an agent’s implicit sense of agency over the effects of an action (Haggard & Tsakiris, 2009). While such applications of the motor approach are still quite common, accumulating evidence has shown that two events can be perceived as temporally shifted towards each other when they are solely causally linked without one of them being an action and the other being its effect (e.g., Borhani et al., 2017; Buehner, 2012, 2015; Kirsch et al., 2019; Ruess et al., 2020). These findings led to the suggestion that perceived causality rather than intentional motor action is the root of temporal binding (*mere causality approach*). The motor approach is further called into question by reports on an absent correlation between temporal binding and explicit agency ratings in action contexts (Dewey & Knoblich, 2014; Obhi & Hall, 2011; Schwarz et al., 2019). Thus, the mechanisms underlying temporal binding have to be subjected to new interpretations beyond being an implicit measure or proxy for agency (Hoerl et al., 2020). The causality approach itself offers an intriguing alternative to the motor approach, but until recently lacked a clear theoretical foundation. It construes intentional binding as one instance of a causal event chain but, on a theoretical level, merely replaces the term of *agency* with *causality*.

Considering temporal binding as an outcome of a multisensory cue-integration process appears to be particularly promising for integrating this phenomenon into a wider conceptual setting. From the perspective of cue integration, two events must be perceived to “belong together” or to be part of one meta-event, at least to a certain degree, in order to have an influence on how other parts of the meta-event are perceived. The integration of two sensory signals in a meta-event is aided by the temporal proximity and the perceived cross-correlation between two events in time. The magnitude of this relation determines the general strength of signal coupling (i.e., of binding). Importantly, when asked to judge the timing of elements of the meta-event, both temporal cues included in this event are combined, and weighted according to their relative precision (Ernst, 2006; Holmes, 2009; Rohde et al., 2016). The strength of coupling is assumed to vary on a continuum from complete fusion of the signals into a single percept to partial integration and complete segregation. In temporal binding settings, participants usually do not fuse both events (action and effect), but obviously apply partial integration expressed in a subjective temporal attraction between the two events that does not completely cover the physical delay between them. In the case of complete fusion, such an integration of distinct multimodal events can be very well explained by a maximum-likelihood estimation model, which results in a more robust multisensory percept compared to each components individual qualities. The same is not necessarily true in partial integration, where the time or space in between the individual cues is also integrated into the multisensory event (e.g., Debats, Ernst, & Heuer, 2017). Nevertheless, we expect that predictions based on relative certainty hold true, even in partial integration.

The multisensory approach to temporal binding has the potential to explain previous findings in a more comprehensive context than the motor approach. Furthermore, it expands the mere causality approach, allowing for clear, quantitative predictions. One critical aspect of the multisensory approach in the current context is that it predicts a different relationship of action and effect binding compared to previous accounts. That is, based on the motor or the mere causality approach both measures may be used interchangeably or in conjunction without changing their conceptual meaning. Therefore, any manipulation of the event chain should influence overall binding, which in turn should be reflected similarly in action as well as in effect binding. The multisensory approach does not preclude the possibility of changes in overall binding capacity, which might result from changes in perceptual precision or perceived causality. However, it also predicts a trade-off between action and effect binding in many situations based on the relative precision (or reliability) of action and effect cues (referred to as *cue certainty* hereafter). For example, when the certainty of the action cue is reduced, while the effect cue remains constant, this should lead to stronger action binding and weaker effect binding and vice versa. These predictions are in line with the common finding that an effect is shifted more strongly towards its cause (effect binding) than the cause is shifted towards its effect (action binding). According to the multisensory approach, this outcome could be due to a higher certainty about the timing of own actions as compared to the timing of external events. Even more strikingly, the magnitude of temporal binding can be manipulated by relatively minor changes in the design, such as delay (Haggard et al., 2002), or the force of a key-press (Cao et al., 2020), which alter neither the action intention nor the causal chain between the action and its effect. Changes in relative cue precision might be responsible for these effects.

Evidence in favor of the multisensory approach comes from experimental designs that actively manipulated the reliability of effect-related signals (Wolpe et al., 2013), or incidentally influenced the reliability of action-related signals. For example, Cao et al. (2020) showed that a light key-press with relatively weak somatosensory feedback is biased more strongly towards an ensuing effect tone than a forceful key-press. The present experiments intended to provide converging evidence from a design that employs a direct manipulation of perceptual precision on both ends of the action-effect episode. This logic was implemented in two experiments, in which we manipulated the temporal certainty of an action as well as the temporal certainty of the ensuing effect alike. In particular, participants used their index finger either to press a key on a keyboard (certain action) or to press against a force sensor placed on a table (uncertain action). In the uncertain action condition, this action was followed by a short beep tone (certain effect). In the certain action condition, either a longer lasting white noise with slow rise and fall (Exp. 1) or a quiet beep tone (Exp. 2) were presented following the action (uncertain effects). Experiment 1 additionally featured a control condition, where a certain action generated a certain effect. We reasoned that exerting pressure on a force sensor press provides less reliable cues for the perception of action timing than a keyboard key-press with tactile on- and offset. In a similar vein, the white noise and the quiet beep tone were assumed to decrease the certainty in the perception of the effect as compared with a well audible beep tone with a clearly defined beginning and end. We tested the validity of these manipulations by comparing variance scores of the certain and uncertain actions, and the certain and uncertain effects in baseline conditions, that is, when their timing was judged in isolation. Reduced perceptual precision of an event is expected to come with higher variances in temporal judgments (see, e.g., Ernst, 2006).^1^

If the different actions and effects are indeed perceived with varying certainty, as intended, the multisensory approach predicts a trade-off between action and effect binding for such a situation. Specifically, when certainty about the timing of the action *decreases* and certainty about the timing of the effect *increases*, the temporal perception of action should be biased strongly towards the effect and the perception of the effect should be less biased toward the action. That is, a stronger action binding and a smaller effect binding is expected for the “uncertain action – certain effect” condition as compared to the “certain action – uncertain effect” condition. Note that the original motor approach and the mere causality approach predict no changes in binding for these critical conditions because action intention as well as the causal chain are constant. Alternatively, if the strength of the causal link is impacted by the current manipulation, they predict similar changes in action and effect binding. Thus finding evidence for the trade-off between action and effect binding would strongly support the multisensory approach. For both experiments, the design and hypotheses as well as the data analysis plan were preregistered prior to data collection (Exp. 1: osf.io/vxn93; Exp. 2: osf.io/29j7p). All statistical analyses of directed hypotheses specified in these documents are reported as one-tailed tests. Raw data and analyses are available online (https://osf.io/spjqh/).

## Experiment 1

### Methods

#### Participants

We collected data of 30 participants at the University of Würzburg and reimbursed them with monetary compensation or partial course credit. The sample size grants a power of 1-β > .99 to detect the effect of tone certainty on action binding in Wolpe et al. (2013). Three participants were excluded (for reasons, see the *Data preprocessing* section). The remaining sample reported a mean age of 31.9 (±12.7) years, six self-identified as male and 21 as female, and one participant reported being left-handed.

#### Apparatus and stimuli

The experiment was programmed with Matlab Version 2016a and the Psychtoolbox plugin. Following the classic temporal binding paradigm, we assessed the subjective timing of actions and following auditory effects. That is, in *operant blocks* participants performed actions and thereby generated auditory effects, whereas they performed key-presses without auditory effects and encountered isolated auditory stimuli in *baseline blocks*. Temporal binding should be evident in later estimates of the key-press in operant blocks as compared to baseline blocks (action binding) and in earlier estimates of the auditory stimulus in operant blocks as compared to baseline blocks (effect binding).

**Fig. 1:**
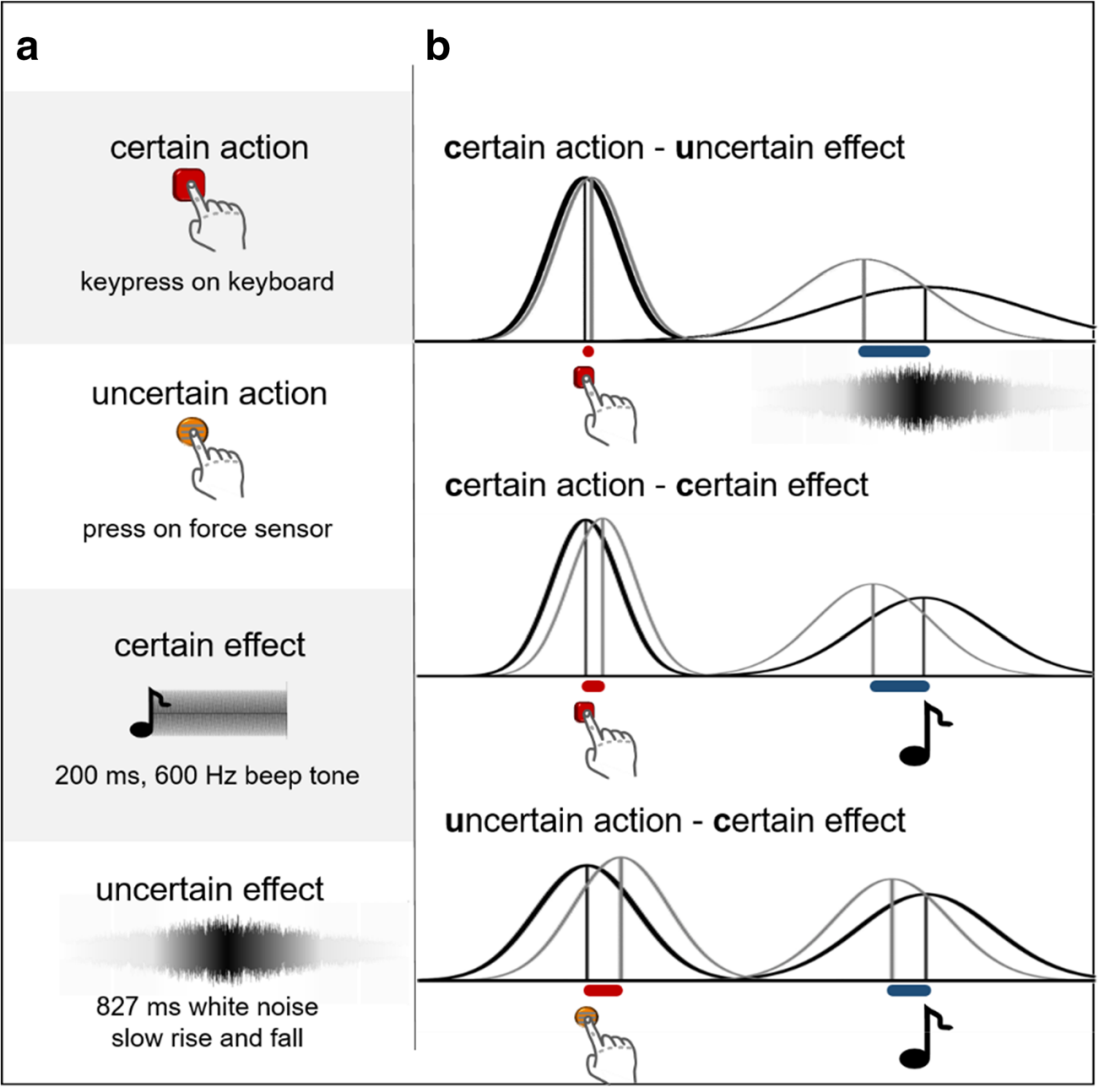
Temporal signals employed in Experiment 1 and related binding predictions. **a** Types of actions and effects in Experiment 1. **b** Predicted binding effects in terms of statistical multisensory cue integration. The idealized likelihood functions illustrate the effect of changing certainty parameters and how they are expected to affect action and effect binding. Black curves show the expected distribution of temporal judgments of actions/effects in isolation, and gray curves show judgments of the same temporal signals in the respective contingent setting. In contrast, the motor and the mere causality approach both predict either no change of binding values between the conditions, if the manipulation has no influence on motor prediction or perceived causality, or similar change of action and effect binding values if there is such an influence

Actions were performed with the left index finger either via key-press on a keyboard (certain action) or on a force sensor fixed on the table (uncertain action). The keyboard was a standard computer keyboard and thus came with clearly defined onsets and offsets for each key-press (3.5-mm travel distance to bottom out key). Presses on the force sensor were accepted if pressure remained within a predefined force range for 50 ms. Participants were asked not to lift their finger between presses. How to perform a successful action via force sensor was explained and briefly trained before the experiment. Effect sounds were played via headphones and were either a 200-ms, 600-Hz beep (certain tone) or 827 ms of white noise that slowly rose and fell (uncertain tone; see Fig. 1A).

During every block, participants saw a Libet clock with ticks at every quarter hour on which they were instructed to estimate the timing of either actions or auditory events. The clock hand began to rotate at the beginning of the trial (taking a full turn every 2 s) and continued to do so for 1.2–1.5 s after the event in question had occurred. Then it stopped moving and jumped to a random position on the clock. Participants were then asked to judge the timing of one element of the trial by moving the clock hand with the arrow keys on the keyboard to the position it had been in at the time of the event, using their right hand. The Libet clock and all written instructions were presented on a 24-in. monitor with a refresh rate of 60 Hz.

#### Design

We implemented two kinds of actions and two kinds of effects that differed in how precisely their timing could be perceived (see Fig. 1A). As is standard in temporal binding experiments, both actions and both effects were once probed in isolation to generate a baseline measure of temporal judgments. Additionally, these actions and effects were combined in three operant conditions (see Fig. 1B).

The “certain action – certain effect” (c-c) condition served as a control condition by replicating typical setups in the literature. Here, a key-press on the keyboard triggered a 200-ms beep tone with a constant delay of 500 ms. In the “uncertain action – certain effect” (u-c) condition, a force sensor press triggered a 200-ms beep tone at a constant delay of 500 ms, whereas in the “certain action – uncertain effect” (c-u) condition, a key-press on the keyboard triggered 827 ms of white noise with a slow rise and fall. The white noise began to rise after a 173-ms delay. All three operant conditions were either presented as action blocks, that is, participants only had to judge the timing of the action in this block, or as effect blocks, in which they only had to judge the timing of the effect. In effect baseline blocks, tones were presented at a random interval of 2–3 s after trial start. In all other blocks, participants were asked to wait at least 1 s before performing their action. Overall, the experiment had ten block types: four baseline blocks, three operant action blocks, and three operant effect blocks. Each block was once presented in a practice phase, which was not entered into data analysis. During the main experiment, every block was presented three times with 15 trials each in an unconstrained randomized order.

#### Data preprocessing

We excluded trials in which participants did not wait for at least a full turn before initiating their actions (4.4%), did not move the Libet clock hand during judgment (2.2% of all trials), and trials in which the temporal judgments deviated more than 2.5 standard deviations (SDs) of the participant’s cell mean (2.1%). Additionally, three participants were excluded: one consistently failed to move the clock hand, one had too many errors (failure to respect the inter-trial interval), and one had too high a variance in their judgments (2.5 SDs above the mean of the full sample). These participant exclusions were not preregistered, but we deemed them preferable to avoid a biased assessment of the results. A re-analysis of the whole sample, including these participants, is available in Appendix A. Furthermore, we computed the estimation error for each block type (judged time – actual time). If the judgment was in the clock half after the actual timing, we assumed a shift forward in time, and if it was in the clock half before the actual timing, we assumed a shift backward in time.^2^

### Results

#### Manipulation check

**Fig. 2:**
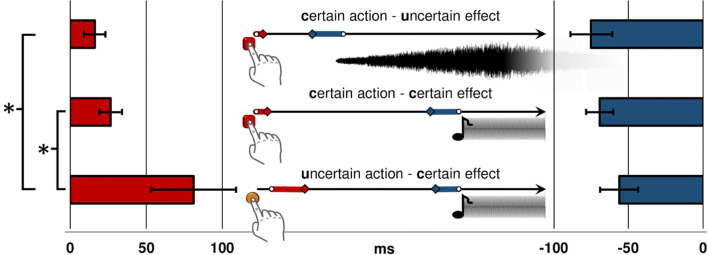
Main results of Experiment 1. Action binding is shown to the left (red bars) and effect binding to the right (blue bars) ± SE of the mean (ms). Centrally, the judged timing in operant blocks of actions (red diamonds) and effects (blue diamonds) as well as judged timing in baseline blocks (white circles) are shown. *p < .05

As a manipulation check, we computed the variance of the estimation errors in baseline blocks, which is assumed to be the inverse of the respective events’ certainty. Baseline blocks of uncertain actions as well as baseline blocks of uncertain effects should thus come with higher variances than certain baseline blocks (see Fig. 2A and Table 1). Indeed, one-tailed paired *t*-tests showed higher variances in uncertain than in certain baseline blocks for actions, *t*(26) = 4.67, *p* < .001, *d* = 0.90, whereas the differences of variance for effects conformed to our hypothesis numerically, but did not reach significance, *t*(26) = 1.70, *p* = .051, *d* = 0.33.

**Table 1:** Mean binding values (ms) (operant – baseline judgment errors) for Experiment 1

		Mean	Paired *t*-test (operant vs. baseline)	Baseline variance
c-u	action binding	16.07	*t*(26) = 2.30, *p* = .015, *d* = 0.44	17048.74
	effect binding	-74.91	*t*(26) = 5.29, *p* < .001, *d* = 1.02	10150.04
c-c	action binding	26.50	*t*(26) = 3.56, *p* = .001, *d* = 0.69	17048.74
	effect binding	-69.16	*t*(26) = 7.66, *p* < .001, *d* = 1.47	8041.49
u-c	action binding	81.21	*t*(26) = 2.91, *p* = .004, *d* = 0.56	91353.43
	effect binding	-56.00	*t*(26) = 4.41, *p* < .001, *d* = 0.85	8041.49

*Note.* Paired *t*-tests are one-tailed and contrast the respective baseline with the operant condition. Conditions: c-u = certain action (keyboard press) and uncertain effect (white noise); c-c = certain action (keyboard press) and certain effect (beep tone); u-c = uncertain action (force sensor press) and certain effect (beep tone). Effect sizes are reported as Cohen’s *d*_z_. Baseline variances are the mean variance of estimation errors in the respective baseline block

#### Main analysis

For the main analysis, we contrasted the judgment error in operant blocks with the judgment error in baseline blocks with paired *t*-tests (one-tailed) to test for the existence of temporal binding. There was significant action and effect binding for all conditions, as shown in Table 1.

Action and effect binding were computed for each type of operant block by subtracting the respective baseline judgment error. Bigger action binding is thus shown by more positive values, whereas effect binding is shown by more negative values. The binding values were entered into a repeated-measures analysis of variance (ANOVA) with the factor certainty-relation (c-u vs. c-c vs. u-c) separately for action judgments and effect judgments. Sphericity could not be assumed for either, and reported *p*-values are based on Greenhouse-Geisser corrected degrees of freedom. Differences between conditions were tested by planned contrasts (see Fig. 2). The ANOVA for action binding showed a significant impact of certainty-relation, *F*(2,52) = 4.25, *p* = .044, η_p_^2^ = 0.14, ε = 0.56. Action binding was strongest when the action was uncertain and the effect certain (u-c), and was significantly smaller when the certainty relation was reversed (Action_u-c vs. Action_c-u), *t*(26) = 2.18, *p* = .039, *d* = 0.42 (two-tailed), but also when only the action certainty increased (Action_u-c vs. Action_c-c), *t*(26) = 1.98, *p* = .029, *d* = 0.38 (one-tailed). The ANOVA for effect binding did not show a significant impact of certainty relation, *F*(2,52) = 1.01, *p* = .357, ε = 0.79, and neither did the planned contrasts (all |*t*|s < 1.13, all *p*s > .135).

#### Follow-up analyses

Based on the marked differences in variances, especially in action blocks, we followed up on the above pre-registered analyses and performed a non-parametric confirmation of the main analysis. That is, we compared action and effect binding in c-u and u-c blocks in a two-tailed paired Wilcoxon signed-rank test (action binding: *Z* = -1.87, *p* = .061; effect binding: *Z* = -1.35, *p* = .178), which did not reach significance.

Nevertheless, the observed pattern of results corroborates the predicted trade-off between action and effect binding, and it may therefore not be appropriate to analyze the two binding scores only in separation. Following this finding, we computed the sum of action and effect binding in all three conditions as a measure of an action-effect binding trade-off (with action binding coming with a positive sign and effect binding coming with a negative sign). If the trade-off account is true, this action-effect sum should be smallest in the “certain action – uncertain effect” (c-u) condition, because the absolute value of action binding is small relative to effect binding, while it should be biggest in the “uncertain action – certain effect” (u-c) condition. On the other hand, if the manipulation influenced action and effect binding in a similar way, as would be predicted by motor or causality accounts, the action-effect sum should not change between conditions. Two-tailed paired *t*-tests show that the sum of both binding scores was bigger in the u-c than in the c-u condition, *t*(26) = 2.75, *p* = .011, *d* = 0.53 (c-u vs. c-c: *t*(26) = 1.26, *p* = .221; c-c vs. u-c: *t*(26) = 2.23, *p* = .034, *d* = 0.43), supporting a trade-off account.

### Discussion

Significant action and effect binding was present in all conditions of Experiment 1. Furthermore, the relationship between action and effect binding strikingly resembled the trade-off predicted by the multisensory approach. A stronger action binding and a descriptively weaker effect binding were observed when the action was comparatively difficult, and the effect rather easy to pinpoint in time (i.e., in the u-c condition) than when the certainty-relation was reversed (i.e., in the c-u condition). Moreover, the results suggest an effect of action certainty on action binding independently from effect certainty, as actions were bound more strongly to the same effect, when they were uncertain as compared to when they were certain.

On the other hand, effect bindings did not differ significantly between conditions, and variances between certain and uncertain effects were not significantly affected by the manipulation either. In addition, participants judged the timing of the uncertain tone very close to its onset, rather than its peak (see Fig. 2 for an illustration of the problem). These observations might indicate an inapt effect manipulation. We thus conducted a second experiment, where we retained our action manipulation, but replaced the effect manipulation with one that was modelled more closely on the manipulation applied in previous work (Wolpe et al., 2013).

## Experiment 2

### Methods

#### Participants

For Experiment 2, we increased our sample size so that we collected data from 40 new participants, of whom five had to be excluded (for reasons, see the *Data preprocessing* section). The remaining sample reported being 26.3 years on average (± 6.5), nine self-identified as male, 26 as female, and one participant reported to be left-handed.

#### Apparatus and stimuli

In Experiment 2 visual information was presented on a 20-in. screen and the uncertainty manipulation of the effect was now implemented by using two tones of different volume (see *Design*). All other specifications were identical to Experiment 1.

#### Design

**Fig. 3:**
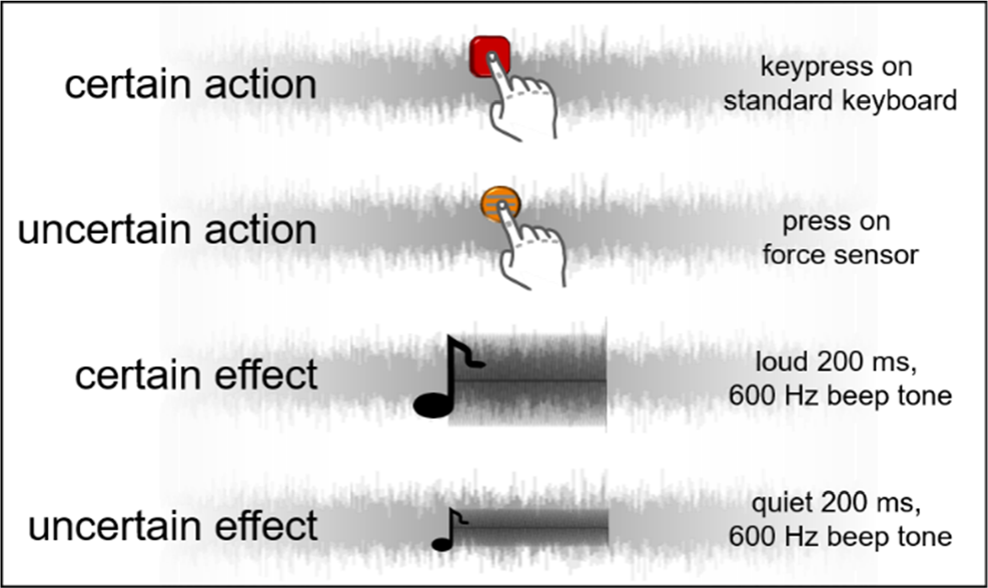
Types of actions and effects in Experiment 2

Experiment 2 closely resembled the first experiment but was subject to two major changes. Firstly, the effect manipulation was adapted to closely resemble one previously applied by Wolpe et al. (2013). Hence, uniform white noise was played during the whole experiment (except breaks) and effects were set as 200-ms 600-Hz beep tones, which were either played loud enough to be easily perceivable over the white noise (certain effect), or were set near perception threshold of each participant (uncertain effect; see Fig. 3). To adjust the volume of the tones individually, participants underwent a simple method of limits procedure (once with ascending and once with descending volume) before the main experiment. The quiet (uncertain) tone volume was set as the mean of the two reported thresholds. The volume of the easily perceptible (certain) tone was set at a fixed value louder than the uncertain tone. None of the individual thresholds exceeded 2.5 SDs from the mean. As a second change to Experiment 1, we dropped the “certain action – certain effect” (c-c) condition. The individual influence of the action manipulation had been shown in Experiment 1 and the influence of the tone manipulation can be assumed, based on Wolpe et al. (2013). Therefore, contrasting the two diametrical conditions appeared to be sufficient to demonstrate the predicted trade-off. Experiment 2 thus had eight block types: four baseline blocks, two operant action blocks, and two operant effect blocks. As before, the experiment started with a practice phase, but due to the reduced conditions, each block of 15 trials was featured five times in the main experiment.

#### Data preprocessing

Data exclusion criteria were similar to Experiment 1, but additionally trials in which participants pressed too strongly on the force key were logged and excluded. Thus, we excluded all trials in which participants did not respect the inter-trial interval, or, unlike in Experiment 1, put too much force on the force key (5.9%), in which they did not move the hand of the Libet clock (3.8%), or in which the temporal judgments exceeded 2.5 SDs of the corresponding cell mean (2.7%). The data of five participants were excluded, as two consistently failed to move the Libet clock hand, one had too many errors (short inter-trial interval/too much pressure on force key), and another two had high variances in their judgments (each > 2.5 SDs from the remaining sample). An analysis of the whole sample is provided in Appendix B.

### Results

#### Manipulation check

To verify the manipulations, we again compared variances of judgments in certain and uncertain baseline blocks. Indeed, in baseline blocks judgments for force sensor presses (uncertain actions) were more variable than for keyboard presses (certain actions), *t*(34) = 2.89, *p* = .003, *d* = 0.49, and variance of judgments for quiet tones (uncertain effect) was higher than for loud tones (certain effect), *t*(34) = 2.72, *p* = .005, *d* = 0.46 (both one-tailed).

#### Main analysis

As before, the judgment error for each block type was computed separately. Then, we compared judgment errors in baseline with those in operant blocks and found participants to show significant action and effect binding in both block types (see Table 2).

**Table 2:** Mean binding values (ms) (operant – baseline judgment errors) for Experiment 2

		Mean	Paired *t*-test (operant vs. baseline)	Baseline variance
c-u	action binding	11.23	*t*(34) = 3.37, *p* < .001, *d* = 0.57	5012.69
	effect binding	-69.56	*t*(34) = 5.91, *p* < .001, *d* = 1.00	16432.97
u-c	action binding	78.25	*t*(34) = 3.85, *p* < .001, *d* = 0.65	27311.54
	effect binding	-53.55	*t*(34) = 4.28, *p* < .001, *d* = 0.72	7134.22

*Note.* Paired *t*-tests are one-tailed and contrast the respective baseline with the operant condition. Conditions: c-u = certain action (keyboard press) and uncertain effect (quiet tone); u-c = uncertain action (force sensor press) and certain effect (loud tone). Effect sizes are reported as Cohen’s *d*_z_. Baseline variances are the mean variance of estimation errors in the respective baseline block

For the rest of the analysis, judgment errors in operant blocks were baseline corrected. One-tailed paired *t*-tests showed greater action binding in the uncertain action – certain effect (u-c) condition than in the certain action – uncertain effect (c-u) condition, *t*(34) = 3.30, *p* = .001, *d* = 0.56. However, as in Experiment 1, the *t*-test showed no significant difference of effect binding between conditions, *t*(34) = 1.24, *p* = .112, even though a trend in the predicted direction was evident.

**Fig. 4:**
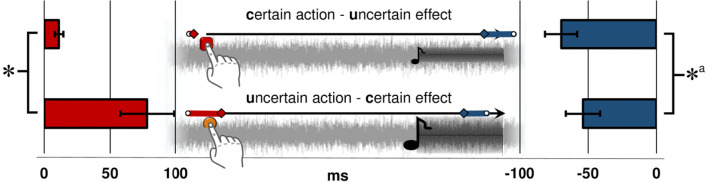
Main results of Experiment 2. Action binding is shown to the left (red bars) and effect binding to the right (blue bars) ± SE of the corrected mean (ms). Centrally, the judged timing in operant blocks of actions (red diamonds) and effects (blue diamonds) as well as judged timing in baseline blocks (white circles) are shown. ^a^Significant only in a non-parametric test. *p < .05

Given the significant variance differences, we compared action and effect binding between conditions again with the same non-parametric test as in Experiment 1 (Wilcoxon signed rank, two-tailed) and found significantly higher ranks of both actions, *Z* = -3.15, *p* = .002, and effects, *Z* = -2.11, *p* = .035, in the uncertain action – certain effect (u-c) than in the certain action – uncertain effect (c-u) condition. Note that regarding action binding, this shows stronger action binding in u-c than in c-u, whereas for effect binding, values are negative and therefore the reverse is true. Following the logic laid out in Experiment 1, we concluded the analysis by testing whether the relationship between action and effect binding would warrant a trade-off account. As before, the sum of action and effect binding differed between conditions, *t*(34) = 2.97, *p* = .005, *d* = 0.50, suggesting a trade-off between the two and therefore supporting a statistical multisensory cue integration view on temporal binding (see Fig. 4).

### Discussion

In Experiment 2, we used an effect manipulation as applied in previous work (Wolpe et al., 2013). The results revealed further evidence for the trade-off between action and effect binding suggested by the multisensory approach. For action judgments, the data replicated a stronger bias for the u-c condition than for the c-u condition as observed in Experiment 1. In addition, in Experiment 2 we also observed the predicted modulation of effect judgments (i.e., stronger effect binding in the c-u condition than in the u-c condition, see the non-parametric test). This outcome supported our suspicion that the lack of differences between effect binding scores in Experiment 1 was due to an inapt effect manipulation.

It is worth noting that Wolpe et al. (2013) reported a very similar pattern of results using only an effect manipulation (i.e., increase in action binding and a decrease in effect binding with an increase in tone loudness). However, the authors argued that the observed variation in effect binding did not arise from cue integration because the effect disappeared when judgment errors in operant conditions were contrasted directly. We do not see compelling reasons for this claim as any direct comparisons without corresponding baseline corrections may be subject to diverse distortions unrelated to temporal attraction of two events and they should therefore not be interpreted in isolation (e.g., Kirsch et al., 2019). We thus suggest that the variation in effect binding observed in Experiment 2 and in the study of Wolpe et al. (2013) is in line with the multisensory perspective, like the differences observed for action binding.

## General discussion

We performed two temporal binding experiments where we manipulated certainty of actions and their effects – that is, how precisely they could be temporally judged. Actions and effects were perceived as temporally shifted towards each other when compared to a condition where they were judged in isolation. Crucially, an action was shifted more towards its effect and the effect was shifted less towards the action (though only descriptively in Exp. 1) when the action was difficult to pinpoint in time while certainty about the effect timing was rather high, compared to a condition where the action was easy to pinpoint in time and certainty about the effect timing was rather low. These results are in line with a multisensory cue integration approach to temporal binding. That is, in the case of an uncertain action and a certain effect, the temporal cues of the effect are weighted stronger and the temporal cues of the action are weighted less than in the case of a certain action and an uncertain effect. Such a statistical integration of both temporal signals results in a stronger bias towards a more reliable cue, and vice versa a weaker bias towards a less reliable cue, and thus predicts the observed trade-off between action and effect binding. This outcome cannot be easily reconciled with the original motor approach or with a mere causality approach. Both accounts would suggest similar results when the intention/ perceived causality does not change between conditions. Though an advocate of the mere causality approach might argue that adding noise to the critical events should decrease the perceived causality, in this case action and effect binding should be affected in a similar way (i.e., both decrease) and the action-effect sum would not be affected in the observed way. Thus, this study adds to the evidence supporting a multisensory cue integration account for temporal binding (Kirsch et al., 2019; Wolpe et al., 2013), rather than assuming a specific mechanism of causality, or even agency.

The present manipulations of cue certainty, especially that of the action certainty, were closely intertwined with task difficulty, however. While there is some evidence that cognitive effort has no influence onto overall temporal binding (Van den Bussche, Alves, Murray, & Hughes, 2020), it still remains to question how far subjective ease of performance is part of the feeling of certainty, or whether the two can be viewed separately. Furthermore, the precise determinants of certainty (i.e., precision of judgments) still need to be pinpointed, possibly by a parametric analysis of a more fine-grained certainty manipulation. Subjective certainty likely goes beyond the objective performance in a temporal judgment task and may not only be globally influenced by individual tendencies and other features of the stimulus, but is also expected to vary on a trial-to-trial basis. Nevertheless, the finding that the subcomponent of certainty considered here (i.e., perceptual precision) had a marked influence on binding values indicates that the hypothesized relationship of certainty and temporal binding indeed exists and might be even stronger than the observed effect. Additionally, it still remains to question why effect binding was less affected by the certainty manipulation than action binding. One possibility is that tones are comparatively hard to judge in time even with no artificially added noise. This assumption appears to be supported by the very common finding that in classic temporal binding settings, effect binding is already much bigger than action binding (e.g., Borhani et al., 2017; Haggard et al., 2002; Schwarz et al., 2019). Just as in the ventriloquist illusion, where the sound is captured by the obvious visual location, the voluntary action might be such a powerful temporal cue that all other manipulations pale in comparison. However, there may also be an alternative mechanism driving effect binding that supersedes or modulates the influence of cue integration on effect binding (Waszak et al., 2012; Wolpe et al., 2013). Note, though, that it is not clear thus far what this alternative mechanism could be. Therefore, we would argue that for now it is reasonable to explore the influence of multisensory cue integration on action and effect binding further, though with a heightened sensitivity regarding the potential of separate mechanisms behind action and effect binding.

## Supplementary Information

ESM 1 (PDF 207 kb)

## Appendix A

For the purpose of this article we reanalyzed data of Experiment 1 and entered all participants into the analysis. Results were similar to those reported in the main text, the only relevant change being (close to) significant effects in the non-parametric reanalysis of the main results.

There was significant action and effect binding for all conditions (see Table 3). An analysis of variance (ANOVA) over all action-binding scores (operant – baseline) showed a main effect of certainty relation, *F*(2,58) = 5.58, *p* = .022, η_p_^2^ = 0.16, ε = 0.55, and planned contrasts showed the expected manipulation when contrasting the diametrical conditions c-u and u-c, *t*(29) = 2.5, *p* = .019, *d* = 0.46 (two-tailed), but also, as in the main text, an individual impact of the action manipulation on action binding (c-c vs. u-c), *t*(29) = 2.5, *p* = .019, *d* = 0.46 (one-tailed). As reported above, the ANOVA on effect-binding scores did not reach significance, *F*(2,58) = 2.43, *p* = .111, ε = 0.79, and neither did any planned contrast (all *t*s < 1.79, all *p*s > .085).

**Table 3:** Mean binding values (ms) (operant – baseline judgment errors) for Experiment 1 without participant exclusions

		Mean	Paired *t*-test (operant vs. baseline)	Baseline variance
c-u	action binding	15.63	*t*(29) = 2.42, *p* = .011, *d* = 0.44	24243.21
	effect binding	-74.63	*t*(29) = 5.74, *p* < .001, *d* = 1.05	18437.32
c-c	action binding	23.20	*t*(29) = 3.06, *p* = .002, *d* = 0.56	24243.21
	effect binding	-61.62	*t*(29) = 5.98, *p* < .001, *d* = 1.09	17802.13
u-c	action binding	94.17	*t*(29) = 3.22, *p* = .002, *d* = 0.59	99841.72
	effect binding	-45.53	*t*(29) = 3.29, *p* = .001, *d* = 0.60	17802.13

*Note.* Paired *t*-tests are one-tailedd and contrast the respective baseline with the operant condition. Conditions: c-u = certain action (keyboard press) and uncertain effect (white noise); c-c = certain action (keyboard press) and certain effect (beep tone); u-c = uncertain action (force sensor press) and certain effect (beep tone). Effect sizes are reported as Cohen’s *d*_z_. Baseline variances are the mean variance of estimation errors in the respective baseline block

Variances of baseline blocks again were significantly different when comparing certain and uncertain actions, *t*(29) = 5.24, *p* < .001, *d* = 0.96, but not uncertain and certain effects, *t*(29) = 0.35, *p* = .366 (both one-tailed). A non-parametric reanalysis of the main results (Wilcoxon signed rank, two-tailed), showed significant differences in ranks for actions (c-u vs. u-c) in the expected direction, Z = -2.23, *p* = .026, and the test also approached significance when contrasting effect bindings (c-u vs. u-c), Z = -1.90, *p* = .057. An analysis of the action-effect sum between conditions again revealed support for a trade-off between action and effect binding (c-u vs. u-c: *t*(29) = 3.10, *p* = .004, *d* = 0.57; c-c vs. u-c: *t*(29) = 2.64, *p* = .013, *d* = 0.48; c-u vs. c-c: *t*(29) = 1.73, *p* = .095, *d* = 0.32).

## Appendix B

The pattern of results of Experiment 2 was replicated when we repeated our analysis on the whole data set, except for the non-parametric reanalysis of the main results, which did not quite reach significance for effect binding without participant exclusions.

Action and effect binding were significant for all conditions (see Table 12) and one-tailed comparisons between conditions showed significant differences for action binding, *t*(39) = 3.89, *p* < .001, *d* = 0.61, but not effect binding, *t*(39) = 0.62, *p* = .269, *d* = 0.10.

**Table 12:** Mean binding values (ms) (operant – baseline judgment errors) for Experiment 2 without participant exclusions

		Mean	Paired *t*-test (operant vs. baseline)	Baseline variances
c-u	action binding	6.84	*t*(39) = 1.93, *p* = .031, *d* = 0.30	5430.80
	effect binding	-58.68	*t*(39) = 3.19, *p* = .001, *d* = 0.50	40634.89
u-c	action binding	79.83	*t*(39) = 4.33, *p* < .001, *d* = 0.68	29996.11
	effect binding	-46.85	*t*(39) = 4.08, *p* < .001, *d* = 0.65	7552.39

*Note.* Paired *t*-tests are one-tailed and contrast the respective baseline with the operant condition. Conditions: c-u = certain action (keyboard press) & uncertain effect (quiet tone); u-c = uncertain action (force sensor press) & certain effect (loud tone). Effect sizes are reported as Cohen’s *d*_z_. Baseline variances are the mean variance of estimation errors in the respective baseline block

Variances for certain and uncertain baseline blocks differed for actions, *t*(39) = 3.44, *p* < .001, *d* = 0.54, and effects, *t*(39) = 2.41, *p* = .010, *d* = 0.38, but, different to the main text, the non-parametric reanalysis showed significant differences between conditions for action bindings, *Z* = -3.79, *p* < .001, and only approached significance for effect bindings, *Z* = -1.87, *p* = .062. The action-effect sum was different between conditions, *t*(39) = 2.78, *p* = .008, *d* = 0.44, and therefore provided evidence for a trade-off account.

## Footnotes

1 Certainty, in this study, is limited to the perceptual precision with which participants are able to judge an event in time. While this performance likely informs the feeling of (un)certainty about an event, it is reasonable to assume that subjective certainty does not always conform to objective performance. It can only be subjective certainty, however, that determines the magnitude of cue integration on a trial-to-trial basis. The measure of perceptual precision can only be a proxy for this.

2 During the review process, we were pointed to an alternative method of analyzing circular data via the von Mises distribution. This method does not require the mentioned centralization of the data. See the Supplementary Material  for a reanalysis of both experiments with this method and corresponding statistical software, (Berens, 2009>). These findings corroborate the pattern observed in the original analysis.
