# Supplementary material for: Temporal binding as multisensory integration: Manipulating perceptual certainty of actions and their effects
Source: Atten Percept Psychophys. 2021 Jun 1;83(8):3135–45. doi: 10.3758/s13414-021-02314-0 (PMC8550101; doi:10.3758/s13414-021-02314-0)
Supplement: Supplementary file 1 — (PDF 207 kb) [file 13414_2021_2314_MOESM1_ESM.pdf]

Supplemental material for

Klaffehn, A. L., Sellmann, F. B., Kirsch, W., Kunde, W., & Pfister, R. (2021), Temporal binding as multisensory integration: Manipulating perceptual certainty of actions and their effects. *Attention, Perception & Psychophysics*. https://doi.org/10.3758/s13414-021-02314-0

Reanalysis of the data with circular analysis methods

During the review process, we decided to reanalyze the data as circular data based on the van Mises Distribution with the CircStat toolbox for Matlab (Berens, 2009)[1]. We recreated our analysis and exclusion criteria in Matlab 2016a and additionally computed the κ parameter for circular dispersion with higher values indicating lower dispersion as compared to the variance computed in the original analyses. In the reanalysis of Experiment 1, five participants were excluded, three of whom had also been removed from the sample in the main analysis. In Experiment 2, seven participants had to be excluded, including four who had been excluded in the original analysis. Suppl. Tables 1, 2, 3, 4, 5, 6, 7 and 8 summarize the main results from this reanalysis side by side with the original results for ease of comparison.

Suppl. Table 1 Experiment 1: Manipulation check

	reanalysis<br>one-tailed paired *t*-test	original analysis<br>one-tailed paired *t*-test
action certain vs. action uncertain	t(24) = 8.00, p < .001, d = 1.60	t(26) = 4.67, p < .001, d = 0.90
effect certain vs. effect uncertain	t(24) = 1.66, p = .055, d = 0.33	t(26) = 1.70, p = .051, d = 0.33

Note. Paired t-tests contrasting each participant's κ parameter (reanalysis) or variance (original analysis) in certain vs. uncertain baseline blocks. Effect sizes are reported as Cohen's d_z.

Suppl. Table 2 Experiment 1: Mean binding values (ms) and test against baseline

		reanalysis			original analysis		
		mean	90% CI<br>(operant vs. baseline)	baseline<br>κ	mean	one-tailed paired *t*-test<br>(operant vs. baseline)	baseline<br>variance
c-u	action binding	16.84	[8.59; 25.09], *d* = 0.69	45.90	16.07	*t*(26) = 2.30, *p* = .015, *d* = 0.44	17048.74
	effect binding	-81.28	[-99.75; -62.81], *d* = 1.50	21.61	-74.91	*t*(26) = 5.29, *p* < .001, *d* = 1.02	10150.04
c-c	action binding	23.02	[13.21; 32.82], *d* = 0.80	45.90	26.50	*t*(26) = 3.56, *p* = .001, *d* = 0.69	17048.74
	effect binding	-67.32	[-80.30; -54.34], *d* = 1.76	25.50	-69.16	*t*(26) = 7.66, *p* < .001, *d* = 1.47	8041.49
u-c	action binding	52.21	[20.69; 83.74], *d* = 0.58	11.51	81.21	*t*(26) = 2.91, *p* = .004, *d* = 0.56	91353.43
	effect binding	-54.65	[-77.85; -31.45], *d* = 0.81	25.50	-56.00	*t*(26) = 4.41, *p* < .001, *d* = 0.85	8041.49

Note. One-tailed tests of binding in operant conditions compared to baseline conditions. Reanalysis columns show the mean differences in estimation error between operant and baseline conditions, the 90% confidence interval for the paired differences and mean κ parameter for the respective baseline conditions. Effect sizes are reported as Cohen's d_z.

[1] Berens, P. (2009). CircStat : A MATLAB toolbox for circular statistics. *Journal of Statistical Software, 31*(10). https://doi.org/10.18637/jss.v031.i10

Suppl. Table 3 Experiment 1: Contrasts between conditions

		reanalysis	original analysis<br>paired *t*-test
c-u vs. c-c	action binding	[-16.85;4.64]1, d = 0.19	t(26) = 1.25, p = .1121, d = 0.24
	effect binding	[-31.49;3.55]1, d = 0.27	t(26) = 0.48, p = .3161, d = 0.22
c-c vs. u-c	action binding	[-59.51;0.16]1, d = 0.35	t(26) = 1.98, p = .0291, d = 0.38
	effect binding	[-32.43;7.79]1, d = 0.21	t(26) = 1.13, p = .1351, d = 0.09
c-u vs. u-c	action binding	[-72.54;1.18], d = 0.41	t(26) = 2.18, p = .039, d = 0.42
	effect binding	[-56.87;4.25], d = 0.36	t(26) = 1.13, p = .135, d = 0.22

Note. Tests for differences of binding between baseline corrected conditions. The reanalysis column contains the confidence interval for the paired difference, the original analysis shows the respective contrasts as paired *t*-tests. Like in the original analysis, the preregistered contrasts of c-u vs. c-c and c-c vs. u-c are reported one-tailed. Effect sizes are reported as Cohen's d_z.

[1] 90% CI/ one-tailed

Suppl. Table 4 Experiment 1: Trade-off account (action-binding + effect binding)

	reanalysis		original analysis	
	mean	95% CI	mean	two-tailed paired *t*-test
c-u vs. c-c	-20.02	[-44.16; 4.12], d = 0.34	-16.17	t(26) = 1.26, p = .221, d = 0.24
c-c vs. u-c	-41.58	[-89.91; 6.74], d =0.37	-67.87	t(26) = 2.23, p = .034, d = 0.43
c-u vs. u-c	-63.10	[-114.20; -11.98], d = 0.53	-84.05	t(26) = 2.75, p = .011, d = 0.53

Note. Effect sizes are reported as Cohen's d_z.

Suppl. Table 5 Experiment 2: Manipulation check

	reanalysis<br>one-tailed paired *t*-test	original analysis<br>one-tailed paired *t*-test
action certain vs. action uncertain	t(32) = 6.67, p < .001, d = 1.16	t(34) = 2.89, p = .003, d = 0.49
effect certain vs. effect uncertain	t(32) = 5.37, p < .001, d = 0.93	t(34) = 2.72, p = .005, d = 0.46

Note. Paired *t*-tests contrasting each participant's κ (reanalysis) or variance (original analysis) in certain vs. uncertain baseline blocks. Effect sizes are reported as Cohen's d_z.

Suppl. Table 6 Experiment 2: Mean binding values (ms) and test against baseline

		reanalysis			original analysis		
		mean	90% CI<br>(operant vs. baseline)	baseline<br>κ	mean	one-tailed paired *t*-test<br>(operant vs. baseline)	baseline<br>variance
c-u	action binding	11.04	[5.78;16.31], d = 0.61	29.56	11.23	t(34) = 3.37, p < .001, d = 0.57	5012.69
	effect binding	-54.47	[-91.45;-17.49], d = 0.46	12.04	-69.56	t(34) = 5.91, p < .001, d = 1.00	16432.97
u-c	action binding	69.36	[38.71;100.02], d = 0.69	10.80	78.25	t(34) = 3.85, p < .001, d = 0.65	27311.54
	effect binding	-42.00	[-57.68;-26.32], d = 0.79	20.24	-53.55	t(34) = 4.28, p < .001, d = 0.72	7134.22

Note. One-tailed tests for significance of binding in operant conditions compared to baseline conditions. Reanalysis columns show the mean differences in estimation error between operant and baseline conditions, the 90% confidence interval for the paired differences and mean κ parameter for the respective baseline conditions. Effect sizes are reported as Cohen's d_z.

Suppl. Table 7 Experiment 2: Contrasts between conditions

		reanalysis<br>90% CI	original analysis<br>one-tailed paired *t*-test
c-u vs. u-c	action binding	[-89.07;-27.17], d = 0.57	t(34) = 3.30, p = .001, d = 0.56
	effect binding	[-48.83;26.15], d = 0.09	t(34) = 1.24, p = .112, d = 0.21

Note. Tests for differences of binding between baseline corrected conditions. The reanalysis column contains the confidence interval for the paired difference, the original analysis shows the respective contrasts as paired *t*-tests.
Effect sizes are reported as Cohen's d_z.

Suppl. Table 8 Experiment 2: Trade-off account (action-binding + effect binding)

	reanalysis		original analysis	
	mean	95% CI	mean	two-tailed paired *t*-test
c-u vs. u-c	-65.35	[-138.70; 8.01], d = 0.38	-83.03	t(34) = 2.97, p = .005, d = 0.50

Note. Circular analysis of the trade-off account only approaches significance. Effect sizes are reported as Cohen's d_z.
